# Supplementary material for: High-Dimensional Bayesian Network Inference From Systems Genetics Data Using Genetic Node Ordering
Source: Front Genet. 2019 Dec 20;10:1196. doi: 10.3389/fgene.2019.01196 (PMC6933017; doi:10.3389/fgene.2019.01196)
Supplement: Supplementary file 1 [file DataSheet_1.pdf]

High-dimensional Bayesian network inference from systems genetics data using genetic node ordering
— Supplementary Information —

Lingfei Wang[1,2,3], Pieter Audenaert[4,5] and Tom Michoel[1,6*]

October 2, 2019

[1] Division of Genetics and Genomics, The Roslin Institute, The University of Edinburgh, Easter Bush, Midlothian EH25 9RG, UK
[2] Broad Institute of Harvard and MIT, Cambridge, MA, 02142, USA
[3] Department of Molecular Biology, Massachusetts General Hospital, Boston, MA 02114, USA
[4] Ghent University - imec, IDLab, Technologiepark 15, 9052 Ghent, Belgium
[5] Bioinformatics Institute Ghent, Ghent University, Ghent, Belgium
[6] Computational Biology Unit, Department of Informatics, University of Bergen, PO Box 7803, 5020 Bergen, Norway
[*] Corresponding author, email: tom.michoel@uib.no

S1 Theoretical background and results

S1.1 Bayesian network primer

We collect here the minimal background on Bayesian networks necessary to make this paper self-contained. For more details and proofs of the statements below, we refer to existing textbooks, for instance [1].

A Bayesian network for a set of continuous random variables $X_1,\dots,X_n$, represented by nodes $1,\dots,n$, is defined by a DAG $\mathscr{G}$ and a joint probability density function that decomposes as

$$p(x_1,\dots,x_n \mid \mathscr{G}) = \prod_{j=1}^{n} p\big(x_j \mid \{x_i \colon i \in \mathrm{Pa}_j\}\big). \tag{S1}$$

We are interested in linear Gaussian networks, which can be defined alternatively by the set of structural equations

$$X_j = \sum_{i\in\mathrm{Pa}_j} \beta_{ij} X_i + \varepsilon_j, \tag{S2}$$

where Pa_j is the set of parent nodes for node j in $\mathscr{G}$ and $\varepsilon_j \sim \mathscr{N}(0,\omega_j^2)$ are mutually independent normally distributed variables. The matrix $\mathbf{B} = (\beta_{ij})$, with $\beta_{ij} = 0$ for $i \notin \mathrm{Pa}_j$, can be regarded as a

weighted adjacency matrix for $\mathscr{G}$. With this notation, the conditional distributions in eq. (S1) satisfy

$$p\big(x_j \mid \{x_i \colon i \in \mathrm{Pa}_j\}\big) = \mathscr{N}\Big(\sum_{i \in \mathrm{Pa}_j} \beta_{ij} x_i, \omega_j^2\Big). \tag{S3}$$

The values of the matrix $\mathbf{B}$ and $\omega_1^2, \dots, \omega_n^2$ are the parameters of the Bayesian network which are to be determined along with the structure of $\mathscr{G}$. The conditional distributions (S3) result in the joint probability density function being multi-variate normal,

$$p(x_1, \dots, x_n) = \prod_{j=1}^{n} p\big(x_j \mid \{x_i \colon i \in \mathrm{Pa}_j\}\big) = \mathscr{N}(0, \Sigma)$$

with inverse covariance matrix

$$\Sigma^{-1} = (\mathbb{1} - \mathbf{B})\Omega^{-1}(\mathbb{1} - \mathbf{B})^T$$

where $\Omega = \mathrm{diag}(\omega_1^2, \dots, \omega_n^2)$. It follows that the gene expression-based term in the log-likelihood of the full model can be written as (up to an additive constant)

$$\mathscr{L}_X \equiv \log p(\mathbf{X} \mid \mathscr{G}) = \frac{m}{2} \log\det \Sigma^{-1} - \frac{1}{2} \mathrm{tr}\big(\Sigma^{-1} \mathbf{X}\mathbf{X}^T\big) \tag{S4}$$

where as before $\mathbf{X} \in \mathbb{R}^{n \times m}$ is the data matrix for n genes in m independent samples. From these basic results, the following can be derived easily:

- For a given $\mathscr{G}$, there exists a suitable ordering of the variables such that $\mathbf{B}$ is lower-triangular. Then $\mathscr{L}_X$ can be written as

$$\mathscr{L}_X = \sum_{j=1}^{n} \left[-\frac{m}{2} \log(\omega_j^2) - \frac{1}{2\omega_j^2} \Big\| X_j - \sum_{i \in \mathrm{Pa}_j} \beta_{ij} X_i \Big\|^2 \right] \tag{S5}$$

  where $X_j \in \mathbb{R}^m$ is the expression data vector for gene j. It follows that the maximum-likelihood parameter values $\hat{\beta}_{ij}$ are the ordinary least-squares linear regression coefficients, $\hat{\omega}_j^2 = \frac{1}{m} \|X_j - \sum_{i \in \mathrm{Pa}_j} \hat{\beta}_{ij} X_i\|^2$ are the residual variances, and $\mathscr{L}_X$ evaluated at these maximum-likelihood values is the log of the total unexplained variance, up to an additive constant

$$\widehat{\mathscr{L}_X} = -\frac{m}{2} \sum_{j=1}^{n} \log(\hat{\omega}_j^2). \tag{S6}$$

- Adding more explanatory variables always reduces the residual variance in linear regression. Hence, for a given $\mathscr{G}$, $\mathscr{L}_X$ is maximized by having all lower-triangular elements $\beta_{ij} \neq 0$. Furthermore, for a nested sequence of DAGs (where one DAG is a subgraph of the next one), $\hat{\mathscr{L}}_X$ as a function of $\mathscr{G}$ is maximized for the fully connected DAG with $n(n-1)/2$ edges[1]. A fully connected DAG $\mathscr{G}$ defines a "topological" node ordering $\prec$ by the relation

$$i \prec j \Leftrightarrow i \in \mathrm{Pa}_j.$$

[1]We use the terminology "fully connected DAG" because *(i)* there exist DAGs with $n(n-1)/2$ edges, and *(ii)* any graph with more than this number of edges contains at least one cycle, that is, is not a DAG.

Equivalently, a node ordering defines a permutation π such that nodes are ordered as $\pi_1 \prec \pi_2 \prec \cdots \prec \pi_n$. Hence eq. (S5) can also be seen as a function on node orderings or permutations, and the maximum-likelihood values are then found by linearly regressing each node on its predecessors (i.e. parents) in the ordering:

$$\mathscr{L}_{X,\pi} = \sum_{j=1}^{n} \left[-\frac{m}{2} \log(\omega_j^2) - \frac{1}{2\omega_j^2} \left\| X_j - \sum_{\pi_i < \pi_j} \beta_{ij} X_i \right\|^2 \right] \tag{S7}$$

- Conversely, eq. (S4), and hence also eq. (S6), is easily seen to be invariant under any reordering of the nodes. Hence no edge directions can be inferred unambiguously from observational expression data without further constraints or information.

S1.2 Pairwise node ordering

To infer Bayesian gene networks, we first consider the log-likelihood score without sparsity constraints,

$$\mathscr{L} \equiv \log P(\mathscr{G} \mid \mathbf{X}, \mathbf{E}) = \log p(\mathbf{X} \mid \mathscr{G}) + \sum_j \sum_{i \in \mathrm{Pa}_j} g_{ij}$$

where it is implicitly understood that the maximum-likelihood parameters are used in $\mathscr{L}_X = \log p(\mathbf{X} \mid \mathscr{G})$. Because $\mathscr{L}_X$ and $\mathscr{L}_P = \sum_j \sum_{i \in \mathrm{Pa}_j} g_{ij}$ are both maximized for fully connected DAGs, and because the value of $\mathscr{L}_X$ is the same for all fully connected DAGs, it follows that to maximize $\mathscr{L}$, we need to find the maximum-weight DAG which maximizes the pairwise score $\mathscr{L}_P$. As stated in the main text, this is an NP-hard problem with no known polynomial approximation algorithms with a strong guaranteed error bound. The greedy algorithm we used is the standard heuristic for this type of problem [2].

S1.3 Sparsity constraints

Using fully connected DAGs leads to overfitting of the expression-based score $\mathscr{L}_X$, particularly in the case where the number of genes n is greater than the number of samples m. The most popular methods for imposing sparsity in Bayesian networks are:

- **Bayesian or Akaike Information Criterion.** The BIC or AIC methods augment the likelihood function $\mathscr{L}_X$ with a term proportional to the number of parameters in the model, i.e. the number of edges $|\mathscr{G}|$ in $\mathscr{G}$ (BIC $= -|\mathscr{G}| \log m$, AIC $= -|\mathscr{G}|$).

- **L1-penalized lasso regression.** In this case, the likelihood $\mathscr{L}_{X,\pi}$ [eq. (S7)] is augmented by a term $\sum_{j=1}^{n} \lambda_j \sum_{\pi_i < \pi_j} |\beta_{ij}|$, such that finding the maximum-likelihood parameters $\hat{\beta}_{ij}$ becomes equivalent to performing a series of independent lasso regressions, one for each node on its predecessors in the ordering π. The extra penalty term can be understood as coming from a double-exponential prior distribution on the parameters β_{ij}.

An under-appreciated drawback of the BIC/AIC in high-dimensional settings is the fact that with a sufficient number of predictors it is possible to reduce ω_j^2 to zero for any gene, and hence make $\mathscr{L}_X$ (S6) arbitrarily large. By concentrating all interactions on one or a few target genes, this can

be achieved while still keeping the BIC/AIC small. Hence in high-dimensional settings, use of the BIC/AIC leads to highly skewed 'all-or-nothing' in-degree distributions, as shown in Figure 2C, unless the maximum allowed number of regulators for each gene is capped at an artificially small number.

Similar problems can occur if lasso regression is used with a fixed λ for all j, because the number of candidate regulators differs greatly among genes that come early or late in the ordering. In [3], a method was proposed where the value of λ_j increases with the order of j, but their scaling could not provide any guarantee for the probability of false positive errors for individual edges in the resultant sparse graph. We used the lassopv variable selection method [4] instead. In brief, for each gene j and for each candidate regulator i of j (i.e. predecessor of j in the ordering π):

- calculate the largest (most stringent) value of λ_j for which i would be selected as a parent of j (i.e. have non-zero lasso regression coefficient);
- calculate the probability (p-value) of a randomly generated predictor having the same or larger 'critical' λ_j.

This results in a set of p-values p_{ij} for all pairs $\pi_i < \pi_j$, which achieve optimal false discovery control, i.e. they can be transformed into q-values q_{ij} by standard FDR correction methods such that if we keep all $q_{ij} \leq \alpha$, the expected FDR is less than α. Moreover for sufficiently small thresholds α, there is a corresponding penalty parameter value $\lambda_j(\alpha)$ such that the set of regulators with p_{ij} (or q_{ij}) less than α is precisely the set of regulators with non-zero lasso regression coefficient [4]. Hence in our method we can use thresholding on the p_{ij} directly to obtain sparse Bayesian networks.

In addition to the lasso regression based method for inducing sparsity, we also considered a simple **thresholding on the pairwise prior information** to obtain a sparse DAG. In the full log-likelihood function, if we set

$$g'_{ij} = \begin{cases} g_{ij} & \text{if } g_{ij} >= \varepsilon \\ 0 & \text{otherwise} \end{cases}$$

then edges with $g_{ij} < \varepsilon$ are automatically excluded from the maximum-likelihood DAG, and the pairwise node ordering procedure will automatically result in a sparse DAG. This method does not provide any guarantee for the false positive control of individual edges in the (multi-variate) Bayesian network beyond what is provided by the pairwise causal inference test used.

S1.4 Summary of terminology

The following terminology is used repeatedly in this paper:

- **"Node ordering"**: a permutation of the nodes.
- **"Edge constraint"**: a set of ordered node pairs $C = \{(i, j)\}$ in a DAG $\mathscr{G}$, that constrains the edges permitted in $\mathscr{G}$ as $\forall\ i \in \mathrm{Pa}_j,\ (i, j) \in C$. Each DAG can be subject to more than one edge constraint.
- **"Topological node ordering"**: a node ordering $\prec$ to a DAG $\mathscr{G}$, that acts as an edge constraint $C \equiv \{(i, j) \mid i \prec j\}$.

- **"Fully connected DAG"**: a DAG $\mathcal{G}$ in which no edge can be added. On a DAG with n nodes and with no edge constraint, it is fully connected if and only if it has $n(n-1)/2$ edges, because the addition of even a single edge is guaranteed to introduce a cycle, that is, $\mathcal{G}$ would cease being a DAG. There is a one-to-one correspondence between node orderings and fully-connected DAGs.

- **"Maximum-weight DAG"**: a DAG that solves the maximum acyclic subgraph problem, identified by its parent sets

$$\text{Pa}^{\max} = \underset{\text{Pa, subjecting to } C}{\text{argmax}} \sum_j \sum_{i \in \text{Pa}_j} g_{ij}$$

  for some set of non-negative prior weights g_{ij}. As discussed in the manuscript, there is no known algorithm for solving the maximum acyclic subgraph problem exactly. For simplicity, we also use the term "maximum-weight DAG" to refer to its heuristic, i.e. the local optima found by the greedy algorithm.

S1.5 Assessment of predictive power for Bayesian networks

The following 5-fold cross-validation algorithm was used to assess the predictive power of different Bayesian network inference methods.

Algorithm S1 Cross-validation of predictive power for Bayesian networks

Require: $M \in R^{n \times m}$ as matrix of normalized expression,
$B(m) \in R^{n \times n}$ as function to infer binary Bayesian network from expression matrix m,
$s(\hat{y}, y)$ as score function (rmse or mlse) of predicted expression $\hat{y}$ given true expression y.

function CROSS-VALIDATION(M, B, s)
  $train\_score, test\_score \leftarrow 0$
  for $i \leftarrow 1$ to 5 **do**
    $train, test \leftarrow$ Random cross-validation split i of training & test data from M
    $\mathcal{G} \leftarrow B(train)$
    for $j \leftarrow 1$ to n **do**
      $model \leftarrow$ Fitted linear model to predict $train_j$ with $train_{\mathcal{G}_{\cdot,j}}$
      $train\_score \leftarrow train\_score + s(model(train_{\mathcal{G}_{\cdot,j}}), train_j)$
      $test\_score \leftarrow test\_score + s(model(test_{\mathcal{G}_{\cdot,j}}), test_j)$
  $train\_score \leftarrow train\_score/5n$
  $test\_score \leftarrow test\_score/5n$
  return $train\_score, test\_score$

References

[1] D Koller and N Friedman. *Probabilistic Graphical Models: Principles and Techniques*. The MIT Press, 2009.

[2] Bernhard Korte and Dirk Hausmann. An analysis of the greedy heuristic for independence systems. In *Annals of Discrete Mathematics*, volume 2, pages 65–74. Elsevier, 1978.

[3] Ali Shojaie and George Michailidis. Penalized likelihood methods for estimation of sparse high-dimensional directed acyclic graphs. *Biometrika*, 97(3):519–538, 2010.

[4] Lingfei Wang and Tom Michoel. Controlling false discoveries in Bayesian gene networks with lasso regression p-values. *arXiv:1701.07011 [q-bio, stat]*, January 2017. arXiv: 1701.07011.

S2 Supplementary figures and table

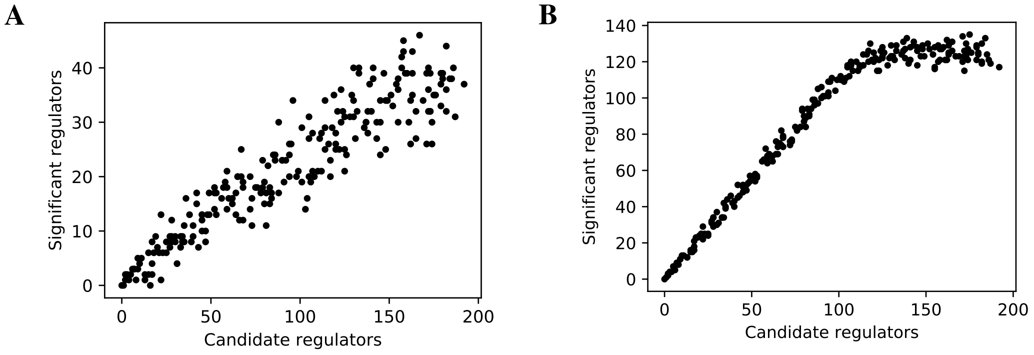

Figure S1: The linearity test of lasso-findr Bayesian networks at 5,000 (**A**) and 20,000 (**B**) significant interactions on DREAM dataset 1.

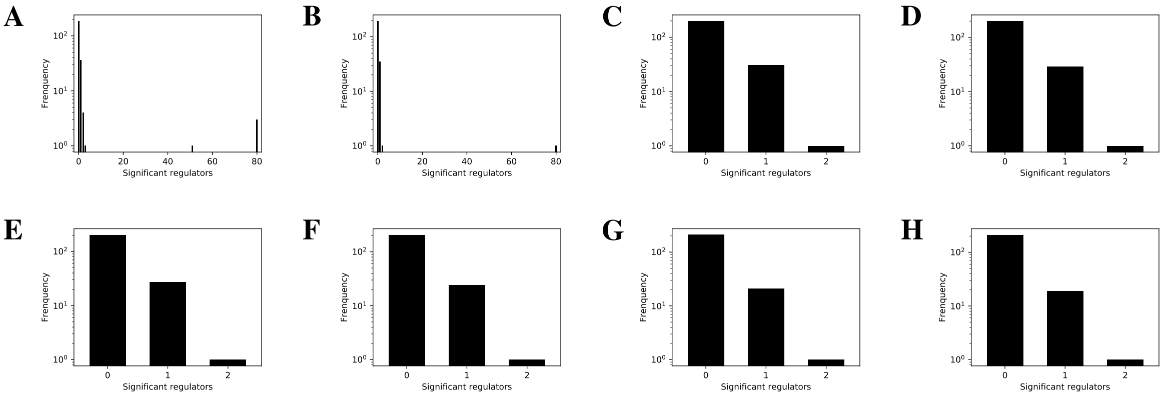

Figure S2: The histogram of significant regulator counts for each target gene in the bnlearn-hc Bayesian networks with AIC penalty 8.5 to 12 (**A** to **H**) and step 0.5 on DREAM dataset 1.

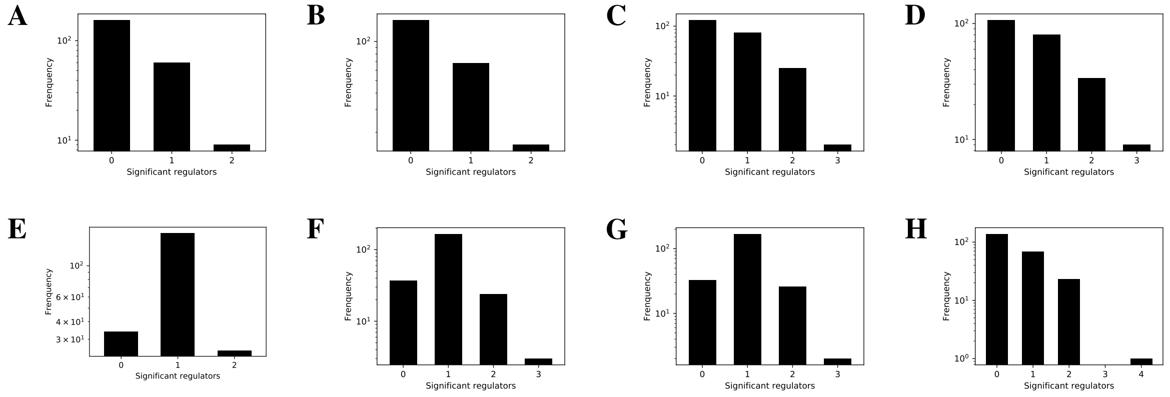

Figure S3: The histogram of significant regulator counts for each target gene in the bnlearn-fi Bayesian networks with nominal type I error rates 0.001, 0.002, 0.005, 0.01, 0.02, 0.03, 0.05, 0.2 (**A** to **H**) on DREAM dataset 1.

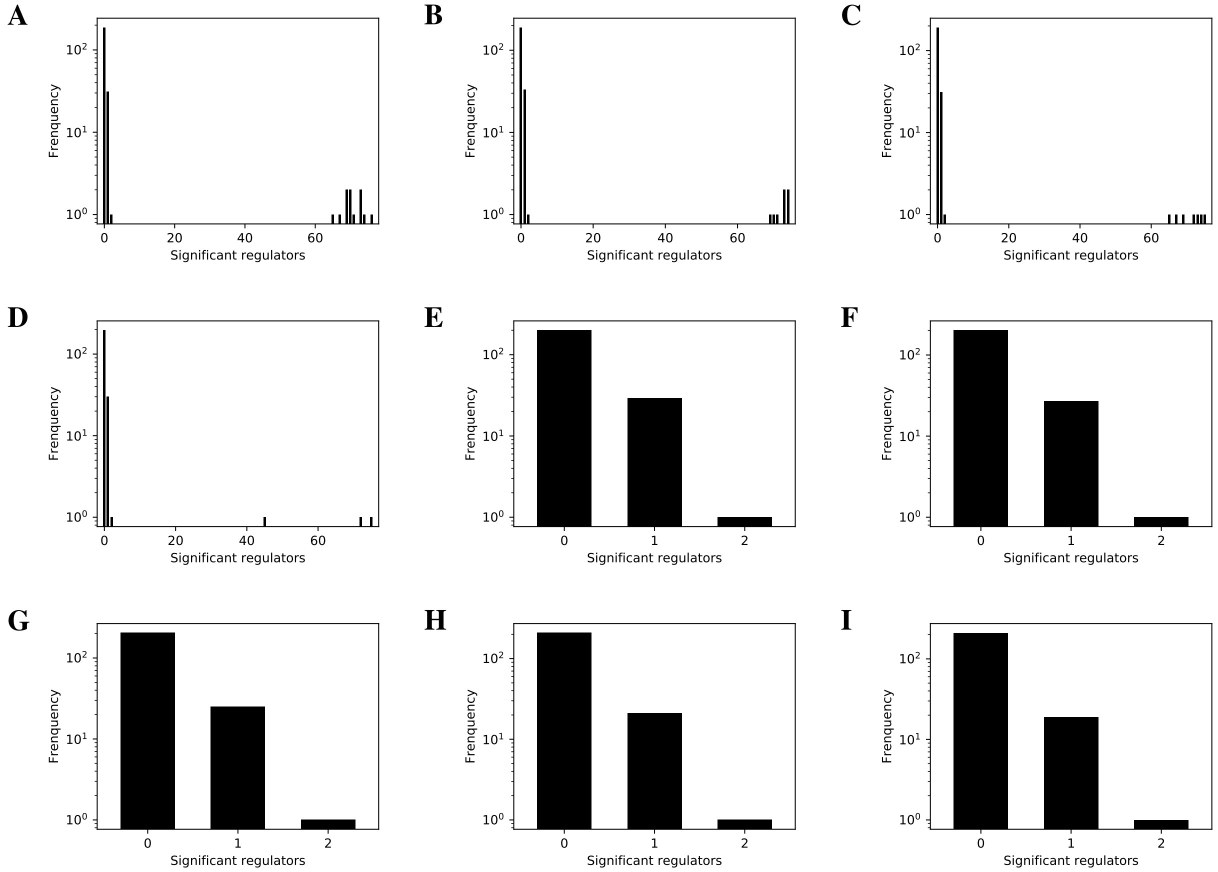

Figure S4: The histogram of significant regulator counts for each target gene in the bnlearn-hc-g Bayesian networks with AIC penalty 9.5 to 13 (**A** to **I**) and step 0.5 on DREAM dataset 1.

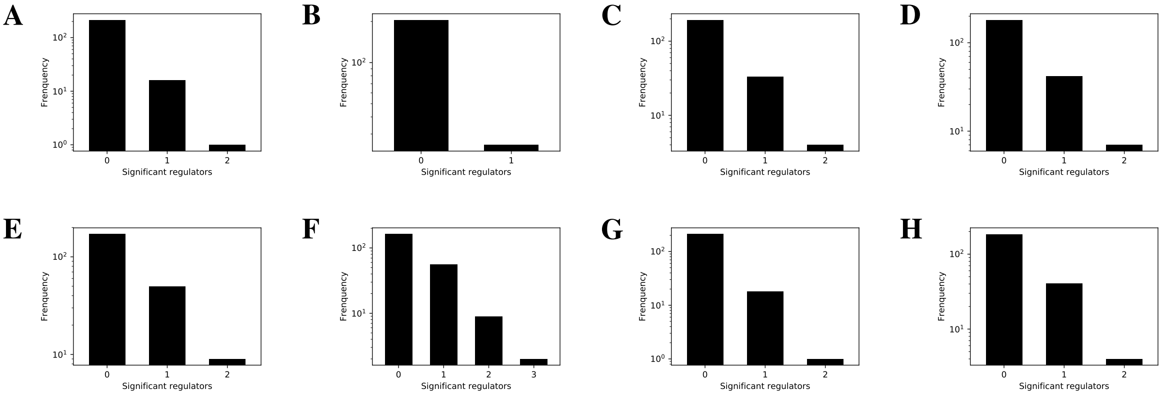

Figure S5: The histogram of significant regulator counts for each target gene in the bnlearn-fi-g Bayesian networks with nominal type I error rates 0.001, 0.002, 0.005, 0.01, 0.02, 0.03, 0.05, 0.2 (**A** to **H**) on DREAM dataset 1.

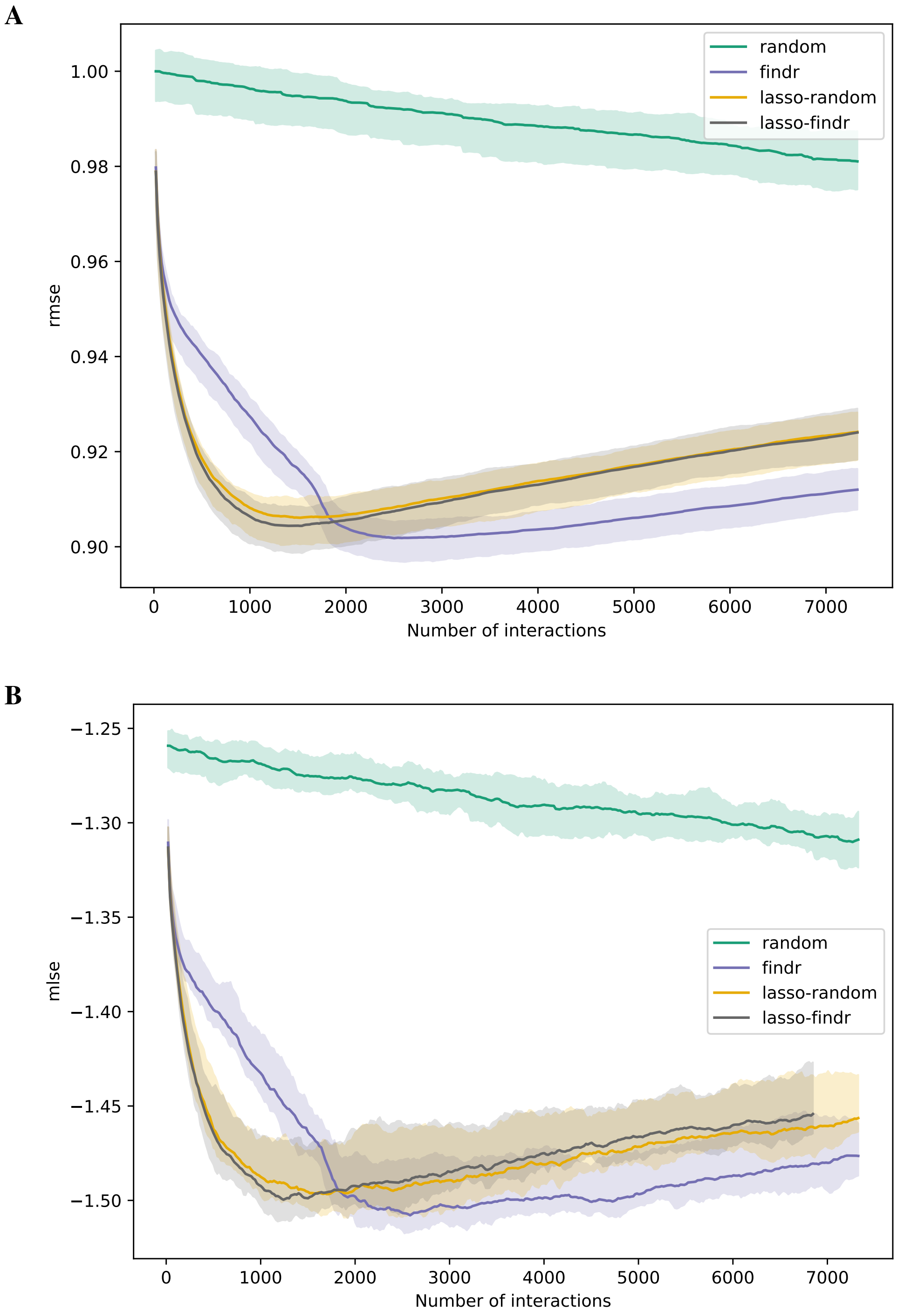

Figure S6: The root mean squared error (rmse, **A**) and mean log squared error (mlse, **B**) in training data are shown as functions of the numbers of predicted interactions in five-fold cross validations using linear regression models. Shades and lines indicate minimum/maximum values and means respectively. DREAM dataset 1 with 999 samples was used.

A

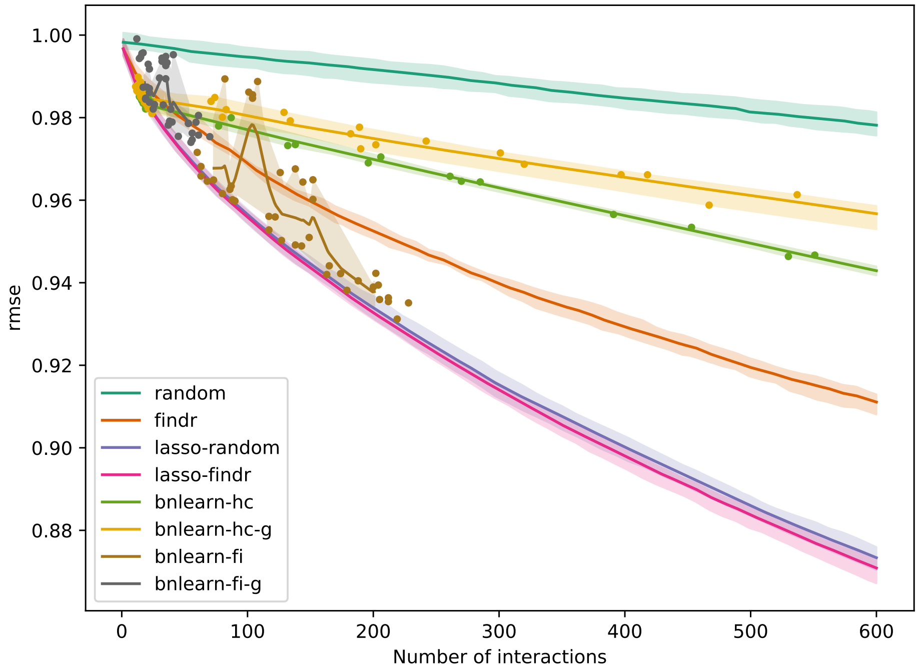

B

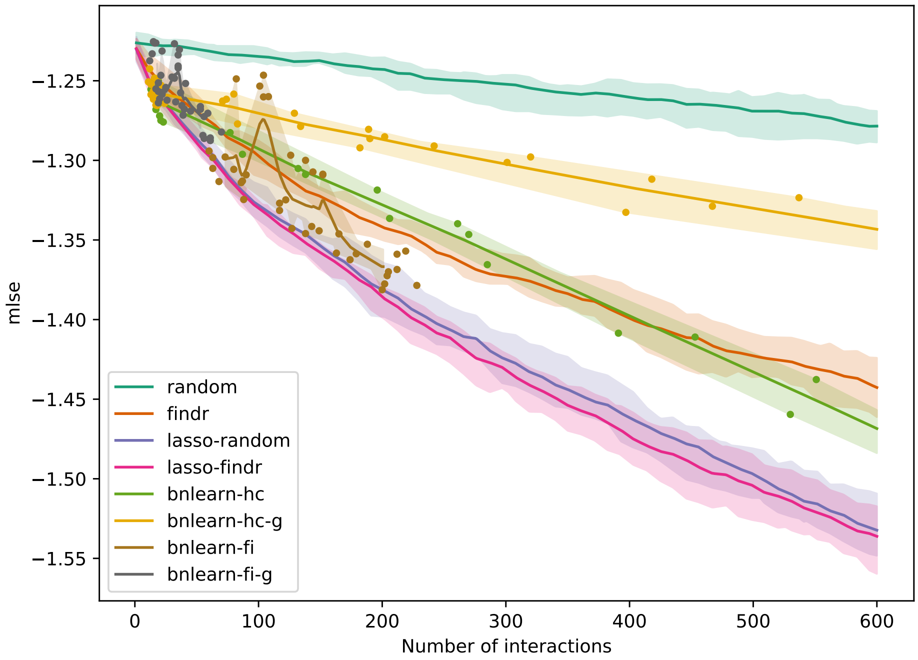

Figure S7: The root mean squared error (rmse, **A**) and mean log squared error (mlse, **B**) in training data are shown as functions of the numbers of predicted interactions in five-fold cross validations using linear regression models. Shades and lines indicate minimum/maximum values and means respectively. Root mean squared errors greater than 1 indicate over-fitting. DREAM dataset 1 with 100 samples was used.

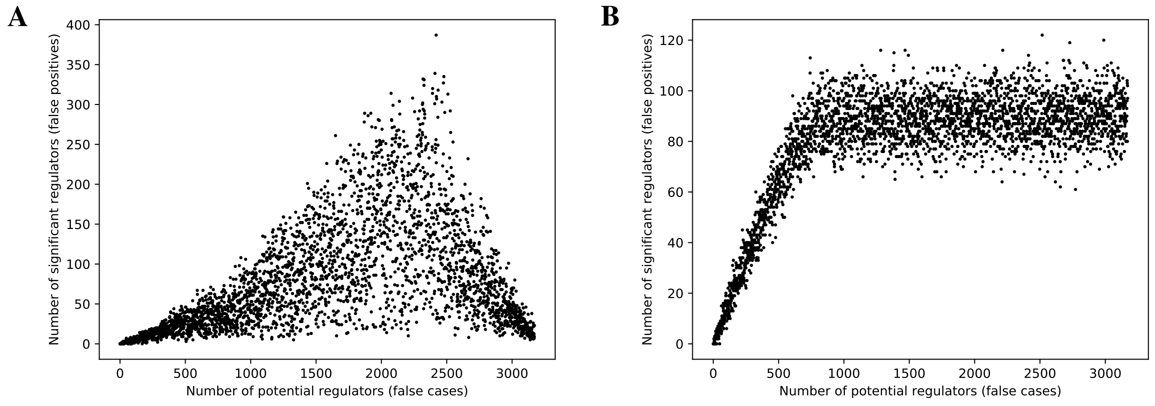

Figure S8: Conversion to Bayesian network from findr's predictions breaks its false discovery control.

Table S1: Main characteristics of Bayesian network inference strategies considered in this paper.

	**Genetic node ordering**	**Score-based**	**Constraint-based**
**Algorithm goal**	Local optimum of log-likelihood	Local optimum of log-likelihood	Graph skeleton representing conditional independences
**Genotype data**	Causal inference between gene pairs	As extra parentless variables	As extra parentless variables
**Edge direction**	From causal inference step	Selected during hill-climbing	From resolution of skeleton constraints
**Search space restriction**	Only BNs compatible with node ordering induced by causal inference	Bounded in-degree	Bounded in-degree
**Sparsity constraint**	Variable selection with FDR control on node ordering	AIC	Nominal Type I error

Table S2: AUPR of network inference on DREAM dataset 1.

findr	0.237
lasso-findr	0.236
lasso-random	0.213
random	0.002
